# The TRAF-interacting protein (TRAIP) is a novel E2F target with peak expression in mitosis

**DOI:** 10.18632/oncotarget.3055

**Published:** 2015-01-02

**Authors:** Christophe Chapard, Daniel Hohl, Marcel Huber

**Affiliations:** ^1^ Service of Dermatology, Lausanne University Hospital, Lausanne, Switzerland

**Keywords:** TRAF-Interacting Protein, TRAIP, E2F transcription factor, promoter regulation, cell cycle

## Abstract

The TRAF-interacting protein (TRAIP) is an E3 ubiquitin ligase required for cell proliferation. TRAIP mRNA is downregulated in human keratinocytes after inhibition of the PI3K/AKT/mTOR signaling. Since E2F transcription factors are downstream of PI3K/AKT/mTOR we investigated whether they regulate TRAIP expression. E2F1 expression significantly increased the TRAIP mRNA level in HeLa cells. Reporter assays with the 1400bp 5′-upstream promoter in HeLa cells and human keratinocytes showed that E2F1-, E2F2- and E2F4-induced upregulation of TRAIP expression is mediated by 168bp upstream of the translation start site. Mutating the E2F binding site within this fragment reduced the E2F1- and E2F2-dependent promoter activities and protein-DNA complex formation in gel shift assays. Abundance of TRAIP mRNA and protein was regulated by the cell cycle with a peak in G2/M. Expression of GFP and TRAIP-GFP demonstrated that TRAIP-GFP protein has a lower steady-state concentration than GFP despite similar mRNA levels. Cycloheximide inhibition experiments indicated that the TRAIP protein has a half-life of around four hours. Therefore, the combination of cell cycle-dependent transcription of the TRAIP gene by E2F and rapid protein degradation leads to cell cycle-dependent expression with a maximum in G2/M. These findings suggest that TRAIP has important functions in mitosis and tumorigenesis.

## INTRODUCTION

The TRAF-interacting protein (TRAIP, TRIP, RNF206) is a 53-kDa protein that contains at its N-terminal end a 55 amino acids long RING domain, followed by a putative coiled-coil domain and a leucine zipper region [1]. TRAIP undergoes auto-ubiquitination in-vitro and, by this criterion, can be considered as functional E3 ubiquitin ligase of the RING class [2]. Historically, TRAIP was reported to interact with the tumor necrosis factor (TNF) receptor-associated factors (TRAFs) [2, 3]. Ectopic expression of TRAIP repressed NF-κB signaling in a RING domain independent manner [2, 3], however, this is most likely a non-physiological function [1]. TRAIP is expressed at low levels in most tissues [3, 4] where it is mainly found in the nucleolus of interphase mammalian cells [5]. The nucleolus is a major nuclear substructure with prominent functions in ribosome formation, the control of the cell cycle and stress responses [6].

TRAIP is required for early mice development since homozygous TRAIP knock-out mice die at embryonic day E6.5/7.5 caused by decreased number of proliferative cells and excessive apoptosis [7]. In a screen for cell cycle regulators, Drosophila maternal effect-lethal mutants affecting the ‘no poles’ (NOPO) gene (CG5140) were identified [8]. This gene encodes a protein with a N-terminal domain showing 47% sequence identity with the RING domain of human TRAIP [8]. The RING domain of one of the NOPO mutants contained a lysine instead of a glutamic acid at position 11, affecting an amino acid highly conserved in TRAIP homologues. Embryos expressing mutant NOPO undergo mitotic arrest during early embryogenesis showing acentrosomal mitotic spindles as most prominent feature [8].

Recently, it has been reported that NOPO/TRAIP interacts with DNA polymerase η which facilitates translesional synthesis after DNA damage [9]. Furthermore, TRAIP is a regulator of the spindle assembly checkpoint (SAC) by regulating MAD2 levels at kinetochores [10]. In addition, TRAIP interacts with CYLD [11] and Syk [5], two tumor suppressors implicated in the formation of skin appendages tumors such as cylindroma, trichoepithelioma and spiradenoma (CYLD) [12, 13] and of melanomas and breast tumors (CYLD and Syk) [14-18].

The TRAIP mRNA level is strongly decreased in primary human epidermal keratinocytes (NHEK) undergoing differentiation induced by high calcium concentration, high cell density or phorbol ester [19], indicating that TRAIP expression is down-regulated when cells exit the cell cycle and undergo differentiation. Inhibition of PI3K or mTOR in proliferating keratinocytes reduced also TRAIP mRNA expression [19]. Knock-down of TRAIP by lentiviral-transduced shRNA in primary keratinocytes resulted in strong inhibition of cell proliferation and a G1/S cell-cycle arrest [19]. These results indicated that TRAIP is implicated in the regulation of cellular proliferation and/or survival.

E2F transcription factors are involved in many ways in the regulation of genes for proliferation, DNA replication, apoptosis and differentiation [20]. E2F DNA-binding sites have been found in the promoters of many genes that are crucial to direct cell cycle progression. Eight proteins (E2F1-E2F8) belong to this family of transcription factors which are subdivided into activators (E2F1-E2F3a), repressor (E2F3b-E2F5) and inhibitors (E2F6-E2F8) [21]. The transcriptional activity of E2F1-E2F5 is regulated by the retinoblastoma protein (pRb) or the related pocket proteins p107 and p130 [22]. To attain their full transcriptional activity and to bind DNA with high affinity, E2F1-E2F6 are required to heterodimerize with dimerization partner (DP) proteins [23].

In this report we show that TRAIP gene expression is induced by E2F1, E2F2 and E2F4 through an E2F binding site located in the 5′upstream promoter region close to the transcription start site. TRAIP mRNA and protein expression correlated with a peak in the G2/M phase suggesting that TRAIP has physiological function(s) in G2/M.

## RESULTS

### E2F1-dependent upregulation of TRAIP expression is inhibited by the retinoblastoma protein

**Fig. 1:**
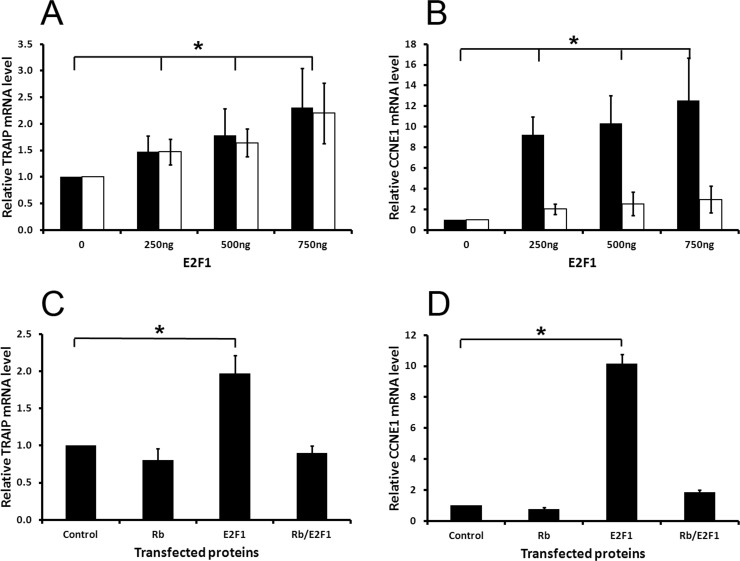
TRAIP mRNA expression is induced by E2F1 but repressed by Rb Relative mRNA levels of TRAIP (A and C) and CCNE1 (B and D) were measured in HeLa cells 24 (black bars) and 48 (white bars) hours after transfection with the indicated plasmids. In the experiments C and D cells were transfected with 400ng pCMV6E2F1 and 600ng pCMV6RB1 plasmid. Control denotes cells transfected with an empty expression vector. RPL13A was used for normalization in quantitative PCR analysis. Results (mean±SD) were derived from 4 independent experiments. Statistical significance of differences between sample and control values was calculated by two-sided paired t-tests, * p<0.05.

Previously, we have shown that inhibitors targeting PI3-Kinase (LY294002) or mTOR (rapamycin) decreased TRAIP gene expression in proliferating keratinocytes [19]. These findings strongly suggested that TRAIP expression was regulated by the PI3K/Akt/mTOR signaling pathway. Since the E2F transcription factors are well known targets of this pathway [24, 25] and, in addition, rapamycin modulates directly or indirectly the transcriptional activity of E2F [24, 26–28], we sought to determine whether TRAIP is a transcriptional target of E2Fs by transfecting HeLa cells with the vector pCMV6E2F1 expressing E2F1 or an empty control vector. After transfection, cells were cultured in low-serum medium (0.25% fetal bovine serum) in order to reduce endogenous E2F activity and to exclude that serum factors affected the results. RNA was isolated 24 and 48 hours posttransfection and the expression of TRAIP and cyclin E1 (CCNE1) was determined by quantitative RT-PCR analysis. The results demonstrated that TRAIP expression was significantly increased by E2F1 in a dose-dependent manner at 24 and 48 hours posttransfection compared to control cells (Fig. 1A). The transcriptional synthesis of CCNE1, a well-known E2F1 target gene [29, 30], was massively upregulated (Fig. 1B), demonstrating the validity of the experimental system used to detect E2F1 targets in HeLa cells.

Previously, it has been shown that the hypo-phosphorylated form of the retinoblastoma (pRB1) protein binds to E2F1 leading to the inhibition of its transcriptional activity [31, 32]. To determine whether the E2F1-dependent activation of TRAIP expression is suppressed by pRB1, HeLa cells were co-transfected with E2F1 and pRB1, and 24 hours post-transfection RNA was isolated for RT-qPCR analysis. The results showed that pRB1 repressed the E2F1-mediated upregulation of TRAIP mRNA expression (Fig. 1C), similarly to the effect of pRB1 on CCNE1 expression (Fig. 1D). In summary, these experiments demonstrated that the expression of the TRAIP gene is antagonistically regulated by E2F1 and pRB1.

### *In silico* analysis of the 5′-upstream TRAIP promoter

**Fig. 2:**
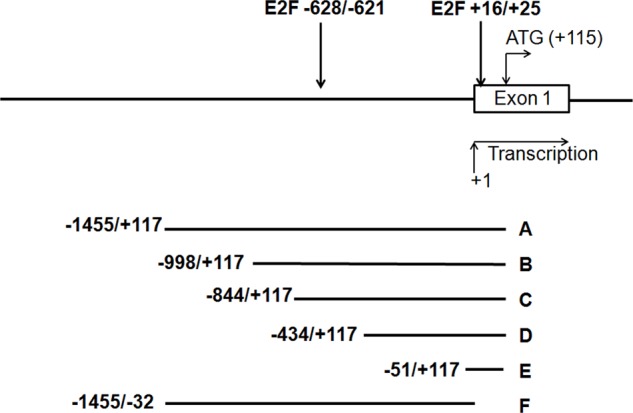
Schematic overview of the 5′ promoter region of the human TRAIP gene The two putative E2F binding sites with their positions relative to the transcription start site are indicated by arrows. The 6 deletion constructs (A-F) which were cloned into the pGL3-Basic luciferase reporter vector are shown below. The transcriptional initiation site is designated as +1 and the nucleotide A of the translation start codon as +115.

*In silico* analysis of the 1.4kbp 5′-upstream region of the human and mouse TRAIP gene detected two conserved regions with similarity to the consensus E2F binding sequence [33]. In the human gene, one of these binding sites is located downstream of the putative TRAIP transcription start site at the position +16 to +25 whereas the second one is found upstream of the transcription start site at position −628 to −621 (Fig. 2). We therefore investigated whether these two E2F binding sites are involved in the control of TRAIP expression by E2F transcription factors.

### Identification of a functional E2F site in the proximal promoter

**Fig. 3:**
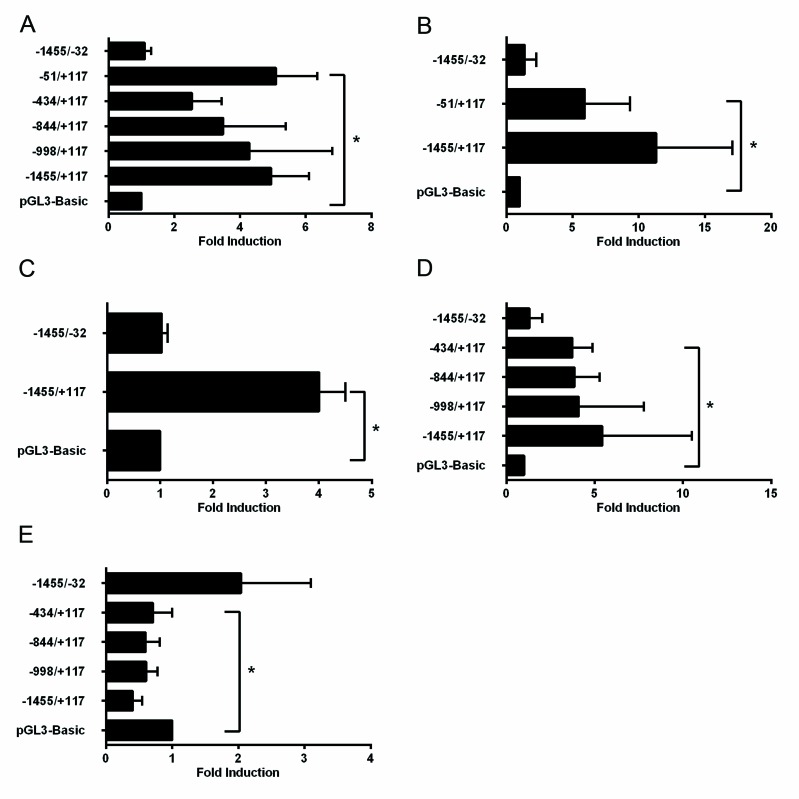
A functional E2F binding site in the TRAIP promoter is located close to the transcription start site Fold induction of reporter activity in HeLa (A-C) cells or NHEK (D-E) transfected with 750ng pCMV6E2F1 (A, D), pCMV6E2F2 (B), pCMVE2F4 (C), or treated with 1μM TPA (E) after transfection of reporter plasmids. Results are expressed as mean±SD from 3-8 experiments performed in duplicates. Statistical significance of differences of reporter activities between promoter plasmids versus pGL3-Basic was calculated by two-sided t-tests, * p<0.05.

Reporter constructs driven by different deletion fragments A-F (Fig. 2) from the 1.4kbp TRAIP 5′-upstream promoter (−1455/+117; positions +1 and +117 denote the transcription start site and the G residue of the start codon, respectively; data according to ENSEMBL ENSG00000183763) were prepared by cloning into the pGL3-Basic vector. HeLa cells were co-transfected with the different reporter constructs and plasmids expressing E2F family members (E2F1, E2F2, or E2F4). The plasmids pcDNA3 and pGL3-Basic were used as control for expression and reporter constructs, respectively. Firefly luciferase activities were adjusted for transfection efficiency with the co-transfected Renilla luciferase activity. The E2F1-, E2F2-, and E2F4-dependent reporter activities with the promoter fragments A-E were comparable and significantly higher than with the pGL3-Basic vector (Fig. 3A–3C). In contrast, the reporter activity of fragment F containing the whole promoter with the exception of 152 bp upstream of the translation initiation codon was indistinguishable from the pGL3-Basic vector with all 3 transcription factors (Fig. 3A–C). These findings argued that the 168 bp region upstream of the translation initiation codon, corresponding to construct E (Fig. 2), contained the functional E2F binding site responsible for E2F1-, E2F2- and E2F4-dependent transactivation of the upstream promoter of the human TRAIP gene in HeLa cells.

Next, we tested the E2F1-dependent reporter activities of the different promoter fragments in primary human keratinocytes (NHEK) cultured in low-calcium medium. The results demonstrated that the fragments A-D had very similar reporter activities which were significantly higher than the basic vector whereas the activity with fragment F was not increased (Fig. 3D). Taken together, we found that only the 168 bp region (−51/+117, Fig. 2) upstream of the translation initiation codon was required for the E2F-induced TRAIP gene expression in different cells. This is in good agreement with the *in silico* identified conserved E2F transcription factor binding site located at position +16 to +25 (Fig. 2). The second putative E2F binding site (−628/−621, Fig. 2) does not seem to be involved in E2F-dependent regulation of TRAIP.

Previously, we have reported that addition of TPA to NHEK cultured in low calcium medium repressed TRAIP expression within 24 hours [19]. Therefore, we investigated whether the promoter activity from the 5′upstream region of the TRAIP gene was modulated by TPA. NHEK were cultured in low calcium medium, transfected with reporter constructs and subsequently treated with 1μM TPA for 24 hours before measuring luciferase activities. The results demonstrated that the reporter activities of the promoter fragments A to D were significantly reduced upon TPA treatment compared to the control vector (Fig. 3E). In contrast, the activity from fragment F was rather increased, albeit not significantly, by TPA. These findings implied that TPA regulated the transcriptional activity of the 5′-upstream part of the TRAIP promoter in a negative manner and localized the TPA-responsive region to the proximal 168 bp fragment of the promoter, similarly as shown above for the E2F-response element.

**Fig. 4:**
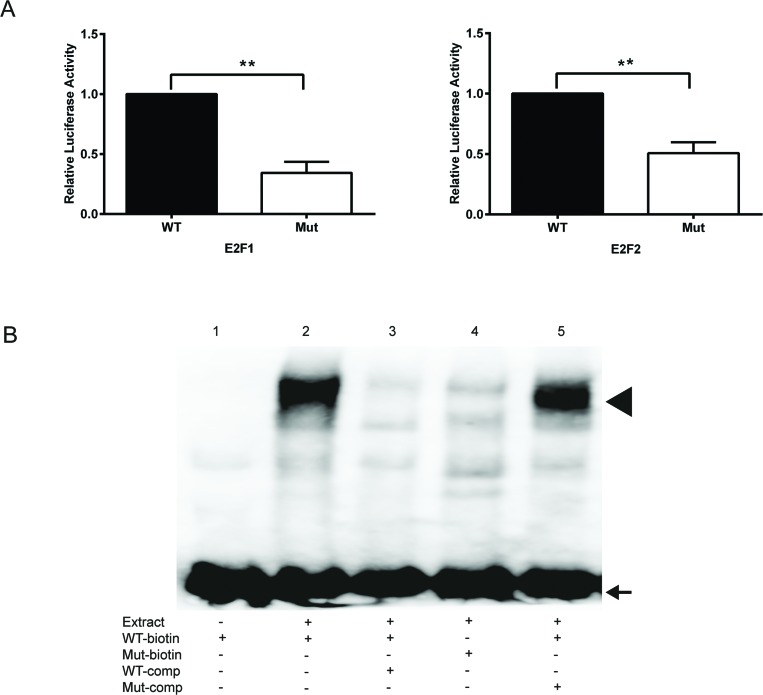
Mutational inactivation of the proximal E2F binding site reduces reporter and EMSA activity (A) Reporter activity of the normal (WT) and the mutated (Mut) TRAIP promoter with E2F1 (left) and E2F2 (right). Results are expressed as mean±SEM from 5 experiments performed in duplicates. Statistical significance of differences of reporter activities between WT and Mut was calculated by two-sided t-tests, ** p<0.01. (B) EMSA experiments with normal (WT) and E2F-mutated (Mut) oligonucleotides using HeLa cell nuclear extracts. Comp; competition with 200-fold molar excess of oligonucleotide added before the biotinylated (biotin) oligonucleotide. Arrow, free oligonucleotide; arrow head, protein-DNA complex.

To further prove the binding of E2F transcription factors to the E2F site at +16 to +25 (Fig. 2), the site was mutated from 5′-TTTGGCTC-3′ to 5′-TTTATCTC-3′ in the full-length promoter (fragment A, Fig. 2). The mutation of the proximal E2F binding site significantly reduced the E2F1- and E2F2-dependent luciferase activities in comparison to the wild type promoter in HeLa cells (Fig. 4A). In electrophoretic mobility shift assays (EMSA) using biotinylated oligonucleotides spanning the proximal E2F binding site (WT) a slower moving protein-DNA complex appeared after incubation with nuclear extracts from proliferating HeLa cells (Fig. 4B, lane 2), which was strongly reduced by competition with 200-fold molar excess of non-biotinylated WT oligonucleotides (Fig. 4B, lane 3). No complex was formed after incubation of HeLa nuclear extracts with biotinylated oligonucleotides containing mutations in the E2F binding site (Mut) (Fig. 4B, lane 4). Furthermore, a 200-fold molar excess of non-biotinylated Mut oligonucleotides was unable to compete with the biotinylated WT oligonucleotides (Fig. 4B, lane 5). In summary, these data strongly suggest the TRAIP expression is regulated by E2F transcription factors through a functional binding site localized in the proximal region of its 5′upstream promoter.

### Endogenous TRAIP expression is cell cycle regulated with a peak in mitosis

**Fig. 5:**
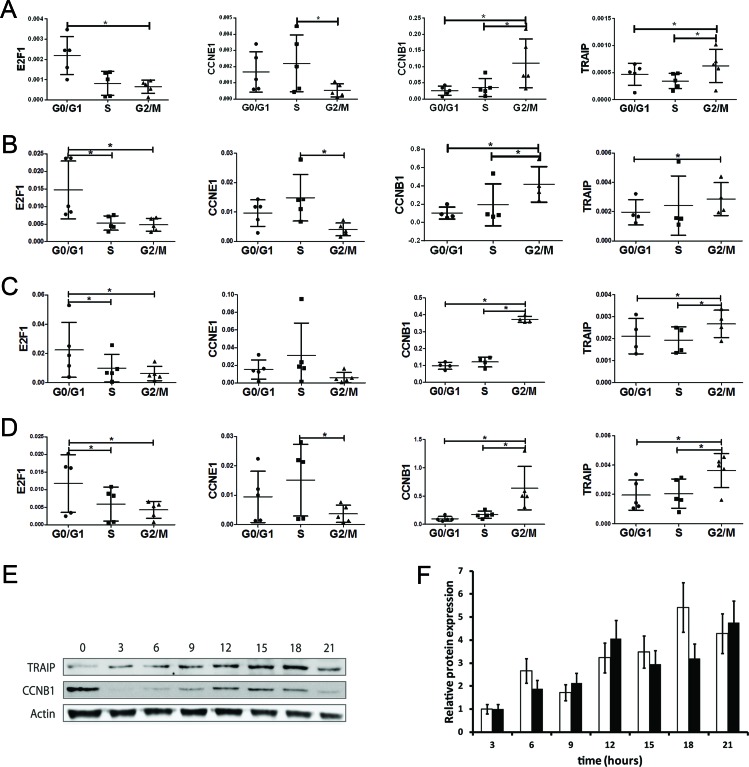
TRAIP is predominantly expressed in the G2/M phase of the cell cycle RNA was extracted from HeLa cells synchronized in the G0/G1, S and G2/M phase as described in Materials and Methods. Relative gene expressions for E2F1, CCNE1, CCNB1 and TRAIP were normalized either with RPL13A (A), GAPDH (B), RPL3 (C) and B2M (D) by quantitative RT-PCR. Results are depicted as scatter plots of 2^ΔCt^ values (mean±SD, 4 independent experiments). Statistical significance of the differences of the 2^ΔCt^ values between the cell cycle phases was calculated by two-sided t-tests; * p<0.05. (E) Western blot analysis of TRAIP and cyclin B1 expression as function of time after release of nocodazole-blocked HeLa cells into normal medium. (F) Protein quantification of cyclin B1 (white bars) and TRAIP (black bars) expression after release from nocodazole block. Results are shown as mean±SD from two experiments using normalization with actin.

Since the TRAIP gene is a target of the cell cycle-dependent transcriptional activity of E2Fs we addressed the question whether endogenous TRAIP gene expression is modulated by the cell cycle. HeLa cells were synchronized either in the G0/G1, S or G2/M phase by serum starvation, double thymidine block or nocodazole treatment, respectively. Analysis of the cell cycle distributions by fluorescence-activated flow cytometry analysis of propidium iodide stained cells revealed that a high percentage of cells was arrested in the desired cell cycle phase: G0/G1, 62.8 ± 2.2%; S, 72.6 ± 5.3%; G2/M, 80.3 ± 3.0%; mean ± SEM, n=4. Endogenous TRAIP mRNA levels in the three cell cycle phases were measured by quantitative RT-PCR analysis using four house-keeping genes (RPL13A, GAPDH, RPL3 and B2M) for normalization. Previous experiments reported that their gene expression levels are minimally regulated during the cell cycle [34]. The results showed that the expression of the TRAIP mRNA was significantly higher in G2/M phase cells compared to G0/G1 phase cells with all genes used for normalization (Fig. 5A–D). The expression of TRAIP in the G2/M phase was also significantly higher than in S phase after normalization with 3 out of 4 house-keeping genes (RPL13A, RPL3 and B2M). To verify whether the experiment was able to predict correctly the cell cycle-dependent expression peak of other genes [34], the mRNA levels of E2F1, CCNE1, and CCNB1 were measured in the same synchronized cell populations. As expected, significantly elevated levels of E2F1, CCNE1, and CCNB1 expression were found in the G0/G1, S, and G2/M phase (Fig. 5A–D), respectively, validating our TRAIP data. To further corroborate these data, HeLa cells were blocked in mitosis by nocodazole treatment, then released into normal medium and protein extracts were prepared every three hours. Quantitative Western blot analysis using actin for normalization of protein loading revealed that TRAIP protein expression was cell cycle regulated with a peak at 15 to 18 hours after the release into nocodazole-free medium (Fig. 5E–F). Again, the expression of the G2/M marker cyclin B1 in the same cell extracts followed a kinetic similar to TRAIP protein levels. Thus, the data indicated that TRAIP gene and protein expression are cell cycle regulated with a maximum in the G2/M phase.

### TRAIP protein has a short half-life

To investigate whether TRAIP protein expression was regulated post-transcriptionally, vectors driving expression of TRAIP-GFP and GFP from a CMV promoter were constructed. TRAIP-GFP was undergoing auto-ubiquitination [2] and localized correctly to the nucleolus [5] as the endogenous protein, indicating that adding GFP at the C-terminal end of TRAIP did not adversely affect the enzymatic activity or the subcellular localization of the fusion protein [10]. HeLa cell lines stably expressing TRAIP-GFP or GFP were obtained by lentiviral infection at equal MOI and hygromycin selection for 11 days. Immunoblot analysis with anti-GFP-antibody revealed that the protein level of TRAIP-GFP was significantly reduced compared to GFP although their steady-state mRNA levels measured by RT-qPCR using GFP-specific primers were very similar (Fig. 5A). This finding suggested that the TRAIP protein level was post-transcriptionally down-regulated, either due to reduced efficiency of mRNA translation or a higher protein degradation rate. To examine this, 293T cells were transiently co-transfected with expression vectors for TRAIP-FLAG and GFP and the decrease of TRAIP-FLAG in the first 6 hours of cycloheximide (50μg/ml) treatment was assessed by immunoblots. GFP or actin were used for normalization since their expressions are more or less stable during the 6 hours of cycloheximide treatment, considering their reported half-lives (26 hours for GFP [35], 48 hours for actin [36, 37]). A representative example of the exponential decay of TRAIP-FLAG is shown in Fig. 5B. The calculation [38] from 4 independent experiments revealed a TRAIP half-life (mean±SD) of 4±1.4 hours or 3.7±1.6 hours using normalization with either GFP or actin, respectively. These findings indicated that TRAIP is an unstable protein explaining the different protein steady-state levels of TRAIP-GFP and GFP in stable HeLa cell lines described above.

## DISCUSSION

Cell cycle progression is a complex physiological process coordinated by a multitude of signaling pathways that are regulated by internal and external cues. Experiments using shRNAs, knockout mice and inhibitors showed that the E3 ubiquitin ligase TRAIP is necessary for cellular proliferation [7, 19]. Since TRAIP expression is regulated by the PI3K/AKT/mTOR pathway [19], which acts upstream of E2F [25–27], we hypothesized that TRAIP is an E2F target gene. Ectopic expression experiments in HeLa showed that TRAIP gene expression is antagonistically regulated by E2F1 and pRB1 (Fig. 1). Indeed, *in silico* analysis of the 5′ upstream region of the human and mouse TRAIP genes revealed two highly conserved putative E2F sites (Fig. 2). Luciferase reporter analysis of the human TRAIP promoter indicated that most, if not all, of the E2F1-, E2F2-, and E2F4-dependent transactivation occurred through the E2F site immediately adjacent to the transcriptional start site (Fig. 3A-E), consistent with the observation that many functional E2F binding sites are located close to the transcription start site [39]. The results from the promoter analysis were not dependent on whether HeLa cells or human keratinocytes were used, indicating that most likely the proximal E2F binding site is important for E2F-dependent TRAIP expression in most tissues. E2F4 was found to activate the TRAIP promoter (Fig. 3C) which might be surprising since this E2F transcription factor is normally thought to function as repressor. However, recent studies indicated that E2F4 can act both as activator and repressor depending on the cellular context and the target gene [40]. The TPA-mediated TRAIP repression in human keratinocytes [19] (Fig. 3E) required only the 168bp fragment containing the proximal E2F site (fragment E, Fig. 2), which would be consistent with data that TPA-mediated activation of PKC inhibits AKT, thus decreasing E2F activity, in mouse keratinocytes [41]. The sequence of the proximal E2F binding site (TTTGGCTC) resembles closely the E2F consensus sequence (TTT G/C G/C CGC) [42]. The functional relevance of the proximal E2F binding site in the upregulation of TRAIP expression was further underlined by comparing wild type and mutated E2F site in reporter assays using the full-length promoter (fragment A) and gel shift experiments (Fig. 4). Inspection of large databases from ChIP-on-chip and ChIP-seq experiments, such as ChIPBase [43] and ENCODE (encodeproject.org), and published data [33, 40, 44–46] provided consistent evidence for the binding of E2Fs to the promoter of the human TRAIP gene in close proximity to the transcription start site using different cell lines and experimental conditions. For example, the repressor complexes E2F4/p130 [44] and E2F4/Sin3a/Sin3b [45] were found to bind to the 5′ upstream TRAIP promoter using ChIP-on-chip analysis in cell cycle-arrested cells and mouse muscle cells undergoing gene silencing during myogenic differentiation. These findings clearly established that TRAIP gene is a novel direct target of E2F1, E2F2 and E2F4. The transactivation occurs through the proximal promoter region containing a bona fide E2F binding site close to the transcription start site. The finding that several E2F transcription factors modulate TRAIP expression in a comparable manner is not surprising since these transcription factors may have redundant regulatory roles in normal and tumor cells [46].

The distal E2F site (TTGGAGG) at the position −628/−621 (Fig. 2) does not seem to play any role in TRAIP promoter activity under the conditions we have tested. Since the similarity to the consensus sequence is lower compared to the proximal site, E2Fs may bind only with low affinity. However, under other conditions the binding site might still be functional, possibly in connection with other transcription factors and co-regulators, since not all functional E2F binding sites match closely the consensus sequence [33].

Deregulation of E2F activity, through overexpression or gene amplification [47–49], or loss of pRB1, through viral proteins or mutations, are frequent events causing different types of human tumors. Since TRAIP gene expression is under control of E2F/pRB1, an increase in E2F transactivation or pRB1 inactivation in tumors could augment the TRAIP level. Mice in which all 3 members of the retinoblastoma family (RB1, p107 and p130) were ablated in liver tissue showed increased Traip expression in hepatocellular carcinoma compared to normal tissue [50]. Analysis of gene expression in tamoxifen-treated Rb^F/F^; K14CreER^TM^; E2F1−/− mouse skin showed a strong decrease in Traip mRNA compared to normal skin [51]. Thus, these *in vivo* results fit well with our *in vitro* data showing antagonistically regulation of TRAIP by E2F1 and RB1. Recent work showed that TRAIP expression is increased in breast epithelial cell lines [5], in breast tumors [52], and in basal cell carcinomas (BCC) [19] with a concomitant decrease in CYLD expression [16, 53], a tumor suppressor interacting with TRAIP [11]. It has been demonstrated in different cultured mammalian cells and Drosophila that activation of the Hedgehog pathway, a hallmark of BCCs [54], leads to the upregulation of cyclins and E2F transcription factors [55–58]. Hence, this most likely provides an explanation for the overexpression of TRAIP in BCCs. Interestingly, CYLD delays G1-S transition by suppressing cyclin D1 expression and E2F activity [59, 60], and influences mitosis [61]. How TRAIP and CYLD functionally interact in cell cycle regulation and cancer development remains to be explored.

The expression of E2F transcription factors and their target genes are modulated during the cell cycle. E2F transcription factors not only play an important role in controlling genes in G1/S phase to coordinate DNA replication, but also regulate genes in G2/M phase encoding proteins important for mitosis [20]. Consistently, TRAIP mRNA and protein expression levels are regulated during the cell cycle reaching a maximum in the G2/M phase (Fig. 5). This is supported by a genome-wide study of gene expression as function of the cell cycle in HeLa cells which listed TRAIP as a periodically expressed gene with a maximum in G2/M [34]. Since the steady-state amount of mRNAs is the combined outcome of synthesis and degradation, the decline in mRNA level after mitosis exit indicates that degradation may be important for TRAIP mRNA regulation during the cell cycle. *In silico* analysis of 3′-UTR of TRAIP mRNA did not reveal any AU-rich elements which are often, but not always, linked to rapid mRNA turnover [62, 63]. Recently, microRNA emerged as important factor regulating mRNA half-life [64]. Indeed, bioinformatic analysis of TRAIP mRNA detected several evolutionary conserved microRNA binding sites in its 3-'UTR. A Traip mRNA half-life of approximately 3.3 hours was found in mouse embryonic stem cells using different culture conditions [65] indicating that the Traip mRNA has an intermediate stability. TRAIP protein abundance followed closely mRNA expression with a peak in G2/M suggesting a strong correlation between mRNA and protein synthesis and degradation. Cycloheximide inhibition experiments demonstrated that TRAIP is a short-lived protein with a half-life around 4 hours although stability analysis using the N-terminal rule [66, 67] predicts that it should be a stable protein with a half-life greater than 20 hours (ProtLifePred, http://protein-n-end-rule.leadhoster.com/). In addition, no good PEST [68] motives (epestfind program in the EMBOSS package, http://emboss.sourceforge.net/) were detected in the TRAIP amino acid sequence.

The two findings that the TRAIP protein is unstable and expressed in a cell cycle-dependent manner as E2F target gene with a peak in the G2/M phase, confer upon TRAIP two properties commonly found for proteins involved in mitosis or cell cycle regulation [69]. During mitosis, numerous molecular factors check for correct mitotic spindle formation and sister chromatid separation to guarantee equal distribution of the genetic material to daughter cells [70]. Interestingly, mitotic defects during syncytial divisions in Drosophila melanogaster embryos harboring RING domain mutants in the NOPO gene, the insect homolog of TRAIP, have been reported [8]. This is consistent with our recent results showing that knock-down of TRAIP in HeLa cells reduced MAD2 recruitment to kinetochores and consequently strongly compromised the activity of the spindle assembly checkpoint, ultimately leading to chromosome alignment and segregation defects [10]. Together with the results reported herein, the conclusion emerges that the TRAIP quantity is tightly regulated at the transcriptional and the posttranslational level to provide high amounts of TRAIP at a specific point during the cell cycle (G2/M) where it is physiologically required to ensure the mitotic progression of cells with faithful distribution of chromosomes to daughter cells.

In summary, we report novel biological properties of TRAIP which is an E3 ubiquitin ligase essential for cellular proliferation. We show that the cellular TRAIP level is tightly regulated by E2F-mediated transactivation and rapid protein turnover to precisely deliver high amounts of TRAIP in the G2/M phase, which is correlating well with its reported functions during mitosis. To understand fully the biological functions of TRAIP, the identification of its physiological substrates will be necessary. Since overexpression of TRAIP has been reported in basal cell carcinomas [19], breast cancer [5, 52] and melanomas (unpublished results), targeting TRAIP or its substrates may provide new possibilities for therapeutic interventions in tumors.

**Fig. 6:**
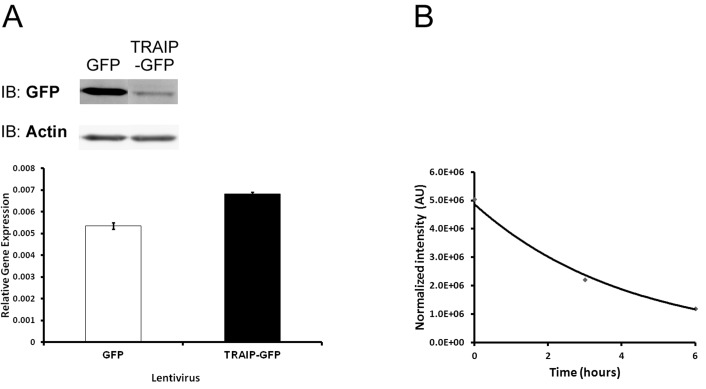
TRAIP protein has a short half-life (A) Immunoblot with anti-GFP antibody (upper panel) and GFP-specific qRT-PCR (lower panel) analysis in HeLa cells infected at identical MOI with lentivirus expressing GFP or TRAIP-GFP at 11 days post-transduction. Actin served as loading control in immunoblot and the GFP mRNA level was normalized with RPL13A. (B) Decay of TRAIP-FLAG protein after addition of cycloheximide (50ug/ml) at time 0. TRAIP quantity was normalized with actin. One representative example from several similar experiments is shown.

## MATERIALS AND METHODS

### Cell culture

HeLa, 293T, and 293FT cells were cultured in 3T3 medium [19]. NHEK from foreskin were cultured until passage 5 as described [19].

### Plasmid constructions

Full-length human TRAIP cDNA was isolated from human keratinocytes by RT-PCR and inserted into the pGEM-T-Easy vector (Promega). All vectors used for the expression of TRAIP and GFP proteins were constructed by PCR amplification and cloning either into pEGFP-N3 (Clontech), pCI (Promega) or pCDH-CMV-MCS-EF1-Hygro (BioCat). Promoter fragments from the 5′-upstream region of the human TRAIP gene were isolated by PCR and cloned into the pGL3-Basic vector (Promega). Mutations in the E2F binding site were introduced by overlapping PCR amplification using Platinum Pfx DNA Polymerase (Invitrogen) and primers containing the desired mutations. All constructs were verified by sequence analysis. Expression plasmids were purchased from Open Biosystems (E2F1; E2F4; and pRB1) or Addgene (E2F2, Addgene plasmid 24226).

### Lentiviral production, titration, and infection

Lentiviral particles were produced in 293FT cells using the packaging vectors pMD2.G (Addgene plasmid 12259) and psPAX2 (Addgene plasmid 12260), and titrated as described previously [19]. HeLa cells were infected with lentiviral supernatant in the presence of 6 μg/ml Polybrene (Millipore) at a multiplicity of infection of 10. Twenty-four hours later, cells were selected for 5 to 7 days with 800μg/ml hygromycin (Calbiochem) until mock-transduced cells were dead.

### Cell cycle synchronization in HeLa cells

HeLa cells were synchronized in G0 phase by 3 days of serum starvation, in S phase by double thymidine block (15h in medium supplemented with 2mM thymidine, 10h release, 15h with 2mM thymidine), and in M phase by 15h treatment with 400ng/ml nocodazole. Synchronized cells were stained with propidium iodide and analyzed for cell cycle distribution by flow cytometry as described [19].

### RNA isolation, synthesis of cDNA and qRT-PCR

RNA was extracted from cultured cells using the RNeasy Mini Kit (Qiagen) and cDNA was produced using the Primescript RT reagent kit (TakaRa). Quantitative PCR analysis was performed with Power SYBRGreen PCR Mastermix (Applied Biosystems) using Quantitect Primer Assay (Qiagen) primers. Relative gene expression was calculated as described [71].

### Luciferase reporter assay

HeLa cells or normal human epidermal keratinocytes (NHEK) were seeded in 24-well plates (2-3×10^4^ cells per well) in 3T3 medium or EpiLife medium with growth supplements, respectively [19]. Cells were transfected the following day with 250ng of the promoter-containing plasmids and 750ng of plasmids encoding transcription factors using jetPRIME (PolyPlus; for HeLa cells) or jetPEI (PolyPlus; for NHEK) as recommended. Cells were co-transfected with 20ng pTK-RL expressing Renilla luciferase to control for transfection efficiency. After transfection, HeLa cells were cultured in low-serum medium (0.25% (v/v) fetal bovine serum) and NHEK in EpiLife medium with growth supplements. Luciferase activities were measured 24 hours later using the Dual Luciferase Assay Kit (Promega). Reporter activity was calculated by dividing the firefly with the Renilla luciferase activity. The ratio of reporter activities for the different promoter constructs was normalized to the pGL3-Basic vector, arbitrarily set to 1 (results expressed as fold induction).

### Nuclear extracts and electrophoretic mobility shift assay

Nuclear extracts from HeLa cells were prepared using the Nuclear and Cytoplasmic Extraction Kit (ThermoScientific). Electrophoretic mobility shift assays were carried out using the LightShift chemiluminescence EMSA kit (ThermoScientific) following the manufacturer's protocol. The following 5′-biotinylated double-stranded oligonucleotides were used as probes: WT, 5′-CCCGCTCCAGGAAGTCGTGCTGCGGAGC CAAATTTG-3′ (the E2F binding site is underlined) and Mut, 5′-CCCGCTCCAGGAAGTCGTGCTGCGAT CATGCGTTTG-3′ (the mutated E2F binding site is underlined). The same oligonucleotides without the biotin were used at 200-fold molar excess for competition experiments. After incubation, the reaction products were resolved on a 5% polyacrylamide gel in 0.5xTBE and transferred to HyBond N+ membrane (Amersham).

### Protein extractions and Western blot analysis

Proteins were extracted from cells by lysis in 1% SDS/phosphate-buffered saline. The following antibodies were used for immunoblots: GFP (Clontech), actin (Sigma), FLAG (Sigma), and cyclin B1 (Cell Signaling Technology). Signals from immunoblots were captured by LAS4000 and quantified by ImageJ.

### Statistical analysis

Statistical analysis was performed with GraphPad Prism 6 and results with p<0.05 were considered significant.

### *In silico* promoter analysis

The 5′upstream region of the human and mouse TRAIP genes were analysed by the rVISTA 2.0 program to find transcription factor binding sites that are phylogenetically conserved [72].

## Abbreviations

B2M: Beta2 microglobulin
CYLD: Cylindromatosis
CCNB1: cyclin B1
CCNE1: cyclin E1
DP: dimerization partner
GAPDH: glyceraldehyde-3-phosphate dehydrogenase
GFP: Green Fluorescent Protein
mTOR: mammalian Target of Rapamycin
NHEK: Normal Human Epidermal Keratinocytes
NOPO: Drosophila melanogaster No Poles gene
PI3K: phosphatidylinositide-3 kinase
PKC: protein kinase C
RING: Really Interesting New Gene
pRB1: retinoblastoma protein
RPL13A: 60S ribosomal protein L13a
RPL3: 60S ribosomal protein L3
SYK: spleen tyrosine kinase
TRAIP: Tumor necrosis factor receptor associated factor (TRAF)-interacting protein
TPA: 12-O-tetradecanoylphorbol-13-acetate.
